# Cardiac endothelial cells and cardiomyocytes alter their communication properties in diabetic mice

**DOI:** 10.1186/s40659-025-00602-9

**Published:** 2025-04-28

**Authors:** Yan Wen, Qing Wang

**Affiliations:** Department of Endocrinology, China-Japan Union Hospital of Jilin University, 126 Xian-tai street, 130033 Changchun, JiLin China

**Keywords:** Diabetes, Cardiomyocytes, Endothelial cells, Cell communication

## Abstract

**Objective:**

We aimed to explore the heterogeneities and communication properties of cardiac CMs and ECs in diabetes.

**Methods:**

GSE213337 dataset was retrieved from NCBI Gene Expression Omnibus, containing the single-cell RNA sequencing data of hearts from the control and streptozotocin-induced diabetic mice. Cell cluster analysis was performed to identify the cell atlas. Data of CMs and ECs were extracted individually for re-cluster analysis, functional enrichment analysis and trajectory analysis. Cell communication analysis was conducted to explore the altered signals and significant ligand-receptor interactions.

**Results:**

Eleven cell types were identified in the heart tissue. CMs were re-clustered into four subclusters, and cluster 4 was dominant in diabetic condition and enriched in cellular energy metabolism processes. ECs were re-clustered into six subclusters, and clusters 2, 4 and 5 were dominant in the diabetic condition and mainly enriched in cellular energy metabolism and lipid transport processes. The cellular communication network was altered in the diabetic heart. ECs dominated the overall signaling and notably increased the ANGPTL and SEMA4 signals in the diabetic heart. Four significant ligand-receptor pairs implicating the two signals contributed to the communication between ECs and other cell types, including Angptl1-(Itga1 + Itgb1), Angptl4-Cdh5, Angptl4-Sdc3, and Sema4a-(Nrp + Plxna2). The ligand Angptl4 engaged in ECs-CMs communication in a paracrine manner.

**Conclusion:**

Single-cell sequencing analysis revealed heterogeneities of ECs and CMs in diabetes, Angptl4-Cdh5 and Angptl4-Sdc3 were involved in the communication between ECs and CMs in diabetes.

## Introduction

Diabetes is a common chronic disease with an estimated global prevalence of 10.5% in 2021, reaching pandemic proportions and continuing to rise rapidly [1]. Diabetes is usually accompanied by cardiovascular complications, which account for at least 50% of all diabetes-related deaths [2]. Diabetic cardiomyopathy (DCM) is a diabetes-specific cardiovascular complication with clinical manifestations of myocardial fibrosis, diastolic dysfunction and heart failure [3]. However, the pathogenesis of DCM is still unclear. Reduced insulin signal transduction, metabolic disorders, oxidative stress and extracellular matrix deposition of myocardia may all lead to DCM [4].

Advancement in single-cell RNA sequencing (scRNA-seq) technology provides an opportunity to map out the cell atlas and characterize the individual cells at the single-cell resolution [5]. Li et al. [6] have applied scRNA-seq to determine the cellular characteristics of diabetic heart tissue. They identified the heterogeneity of the fibroblasts and determined a specific fibroblast cluster that plays an important role in cardiac fibrosis in diabetes. Their study provides new insights into the cellular level of muscle fibrosis at the heart of DCM through scRNA-seq.

Except for the fibroblasts, cardiomyocytes (CMs) and cardiac microvascular endothelial cells (ECs) are the major and vital components of the heart [7]. CMs maintain cardiac contraction mainly through electrophysiological conduction [8], ECs are a layer of cells lining the inner surface of the heart that regulates heart contraction and blood flow, providing oxygen and nutrients to CMs [9]. ECs can control the contractility of CMs by releasing nitric oxide (NO), endothelin-1 (ET-1), CMs and growth factors. Both ECs and CMs expressed Cx37 connexin in direct intercellular contact [10]. In the condition of diabetes, hyperglycemia will limit the utilization of glucose by CMs, produce excessive fatty acid (FA), and damage cardiomyocytes. At the same time, it can increase the permeability of ECs and impair the ability to induce vasodilation [11]. The inflammatory marker TNF-α in diabetic cardiomyopathy reduces the bioavailability of NO released by EC, leading to the contractility of CMs. The significance of endothelium-cardiomyocyte crosstalk as a potential therapeutic target for cardiometabolic diseases was demonstrated [12]. However, information on the cardiac ECs-CMs interaction under the diabetic condition is limited, which requires further research to characterize their communication properties with the aim of developing novel interventions and therapeutic strategies.

In the present study, we applied a different approach by leveraging the available scRNA-seq data. We focused on the characteristics and heterogeneities of CMs and ECs in the diabetic heart, particularly their differentiation trajectories and biological functions in the transition from the normal condition to the diabetic condition and revealed the altered cellular communication and signaling between the two cell types in diabetes. Furthermore, the present study has improved the understanding of the phenotypic changes of CMs and ECs and provided further data on the EC-CM cross-talk in diabetes.

## Methods

### Data collection and preprocessing

The gene expression profile GSE213337 was retrieved from the NCBI Gene Expression Omnibus database. The dataset was produced by Li et al., which contains the sc-RNA-seq data of hearts from the control and streptozotocin (STZ)-induced diabetic mice [6]. Briefly, male C57BL/6 mice (22–25 g, 8 weeks old ) were injected with 150 mg/kg of STZ intraperitoneally to construct a diabetic model. The glucose level above 16.7 mmol/L indicated that the diabetic model was successfully completed, and the equal volume of sodium citrate was injected intraperitoneally as control. The mice were sacrificed eight weeks after injection, and the hearts of mice were extracted. The single-cell suspension was obtained by enzyme digestion, and scRNA-Seq was performed.

Data were read into the R software (version 4.1.0) using the Seurat package (version 4.1.0) for preprocessing. The cells with > 200 genes and < 30% mitochondrial genes were screened for further analysis. After log-transformation, the top 2000 highly variable genes were calculated using the FindVariableGenes function. The RunPCA function was utilized for linear dimension reduction, and the Harmony package (version 0.1.0) was used to remove the batch effects. The top 30 principal components were used to calculate the nearest-neighbor distance by “FindNeighbors”.

Moreover, UMAP was applied for nonlinear dimension reduction, “DoubletFinder” package (version 2.0.3) to remove double cells. Finally, the data filtering was performed and cells with Nfeature RNA greater than 200 and nCount RNA less than 20,000 were left for analysis.

### Cell cluster analysis

The FindClusters function was adopted for cell clustering with a resolution of 0.5. The FindAllMarker function was utilized to calculate the differentially expressed genes among the diverse cell subclusters (min.pct = 0.25, logfc.threshold = 0.25, only.pos = TRUE).

The data of the CMs and ECs were extracted for reclustering and analysis, respectively. The FindClusters function was adopted for cell clustering with a resolution of 0.3. A functional enrichment analysis was performed by using the clusterProfiler package (version 4.2.2). The Monocle2 package (version 2.22.0) was applied for the trajectory analysis.

Use the “newCellDataSet” function to generate an object for trajectory analysis (expressionFamily = negbinomial. size). Only genes with a mean expression value greater than 0.1 and expressed in at least 10 cells were used for the trajectory analysis. The “differentialGeneTest” function was used to identify the differentially expressed genes among the cell subsets, and the genes with q-value < 0.01 were used for dimension reduction. We use the “reduceDimension” function to reduce the dimension, with parameters set to reduction_method= “DDRTree” and max_components = 2. Use “plot_cell_trajectory” to sort and visualize the cells. We used “differentialGeneTest” function parameter set to fullModelFormulaStr= “~sm.ns(Pseudotime)” to calculate genes changed with pseudotime (q-value < 1e-10).

### Cell communication analysis

The CellChat package (version 1.1.3) was utilized for cell communication analysis to investigate the significant signaling pathways and ligand-receptor interactions. We used the “createCellChat” function to build CellChat objects and used CellChatDB.mouse as the ligand and receptor database to select the interaction type of “Cell-Cell Contact”. We used “getMaxWeight” to identify the number of cell interactions compared with the network map.

### Construction of DM mice model and validation of novel genes

To further validate the novel genes in both the ECs and CMs, the diabetic model was constructed according to the report from Li et al. [6]. Male mice (C57, 22–25 g, 8 weeks old) were purchased from Meixuan Biological Co., Ltd (Shanghai, China). The animal studies obtained the approval from the China-Japan Union Hospital of Jilin University (SY202311022). Subsequently, mice from the control and Diabetes mellitus (DM) groups (*n* = 6) were sacrificed eight weeks after diabetic conduction. Based on the study by Li et al. [6], all the mice were euthanized and then the hearts were carefully cut off and saved in a tissue storage solution. Moreover, real-time quantitative PCR (qRT-PCR) analysis was performed to investigate the mRNA expression of the novel genes and immunofluorescence for their protein expression in the heart.

### Cell culture

ECs and CMs (Procell, Wuhan, China) were purchased and cultured in special medium. ECs were cultured alone as the control group, and ECs and CMs were co-cultured as the experimental group. After the cells reached 90% confluence, both groups of cells were cultured in medium containing 16.7mM glucose (high glucose, HG) at 37 ° C and 5% CO_2_ for 24 h. The cells were then harvested, and Western blot was used to detect the expression of the ligand receptor.

### Quantitative real-time polymerase chain reaction

Cells were lysed using Trizol (Invitrogen, Carlsbad, USA) and RNA was extracted. The extracted, total RNA was reverse transcribed into cDNA using an Ex Script-TM RT kit (Takara, Kusatsu, Japan), mRNA was detected by an CFX96 TOUCH real-time PCR machine (Bio-Rad, Hercules, USA) using 2x SYBR Green qPCR premix, and the degree of mRNA in each sample was normalized to GAPDH RNA levels. The primers were as follows: GAPDH-F: CTTTGGTATCGTGGAAGGACTC, GAPDH-R: GTAGAGGCAGGGATGATGTTCT; Angptl4-F: GGGACGAGATGAATGTCCT, Angptl4-R: CTTGAGTTGTGTCTGCAGG; Cdh5-F: ATTGGATTTGGAACCAGATGC, Cdh5-R: CGCTTGACTTGATCTTGCC; Sdc3-F: GCTCGTAGCTGTGATTGTG, Sdc3-R: CTTCATACGATAGATGAGCAGTG.

### Immunofluorescence

The expressions of Angptl4, Cdh5 and Sdc3 in myocardium of mice were detected by immunofluorescence staining. After deparaffinization, rehydration, and antigen repair, blocking treatments were performed for 30 min. The slides were incubated with Angptl4(1:1000, ABconal, Wuhan, China), Cdh5(1:1000, ABconal, Wuhan, China) and Sdc3(1:2000, ABconal, Wuhan, China).at 4 °C overnight, then incubated with horseradish peroxidase (HRP) -conjugated secondary antibody for 1 h. Nuclei were counterstained with DAPI. Pictures were collected using a fluorescence microscope after sealing the slides.

### Western blot

Cells were lysed in RIPA buffer (Beyotime, Shanghai, China) and centrifuged at 12,000 rpm for 30 min at 4 °C for protein isolation and collection. Protein concentrations were measured using the BCA Protein Assay kit (Beyotime, Shanghai, China). Samples were subjected to electrophoretic transmembrane and incubated with primary antibodies overnight at 4 °C (Angptl4 1:1000, Cdh5 1:1000, Sdc3 1:2000). After 2 h of membrane with secondary antibody (1:5000), color was developed using DAB color development solution and visualized by Chemidoc xrs gel imaging system (Bio-Rad, Hercules, USA). Quantitative analysis was processed by Image J software.

## Results

### Cellular composition of the mice hearts

**Fig. 1:**
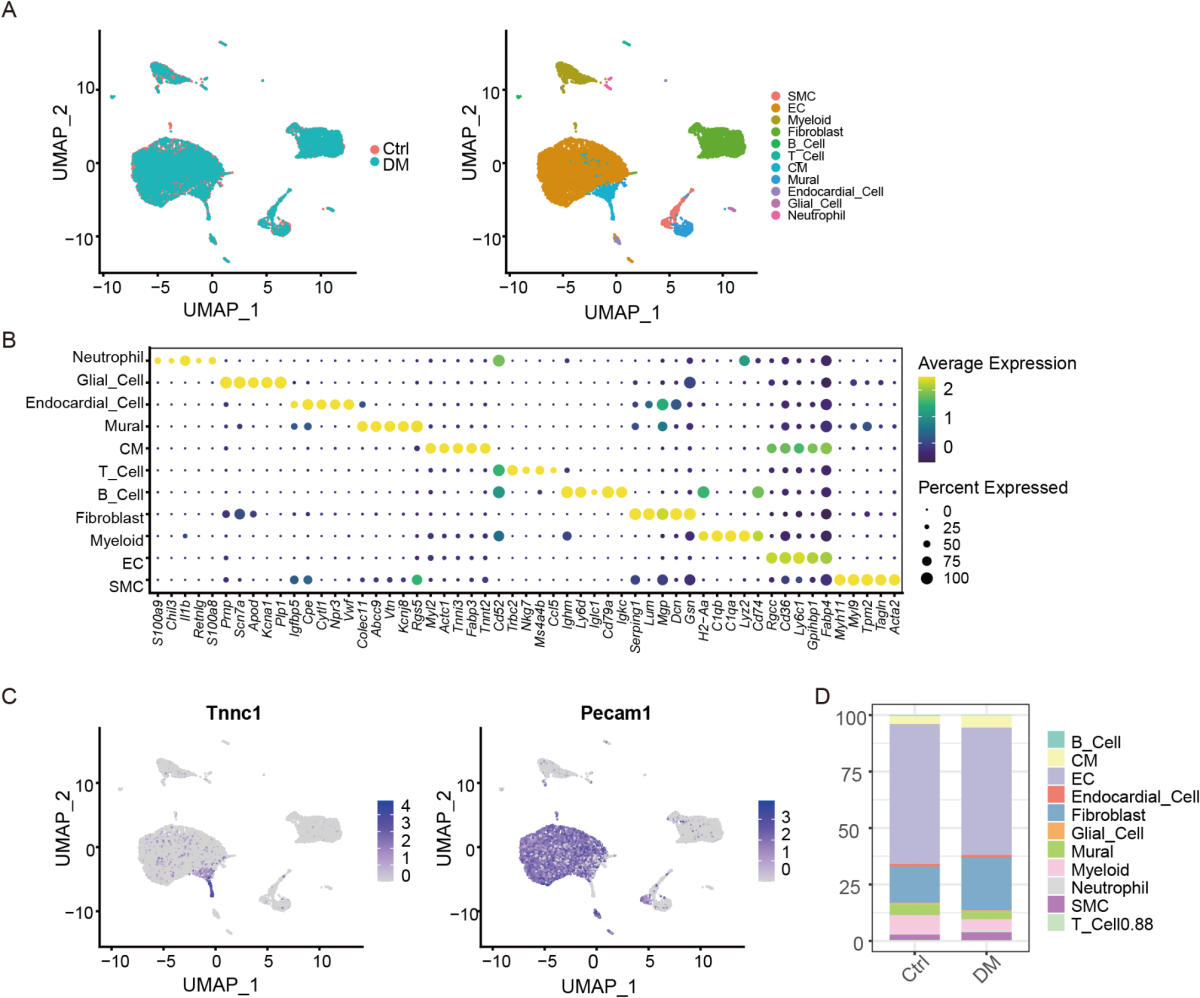
Cellular composition of the heart samples from the control and diabetic mice. (**A**) Eleven cell types were identified based on the highly expressed cell-specific gene markers. (**B**) The dotplot shows the highly expressed genes of each cell subpopulation. (**C**) The gene markers Tnnc1 and Pecam1 showed the cellular localization of CM and EC, respectively. (**D**) The proportions of the cell types were altered in control and diabetic hearts

A total of 11,820 cells were obtained after data prepocessing. The cell profiles of the control and diabetic hearts were different, and 11 cell types were identified (Fig. 1A) based on the highly expressed cell-specific gene markers (Fig. 1B), including smooth muscle cell (SMC), EC, myeloid cells, fibroblasts, B cells, T cells, CMs, mural cells, endocardial cells, glial cells, and neutrophils. The markers Tnnc1 and Pecam1 showed the cellular localization of CMs and ECs, respectively (Fig. 1C). The ECs were the major cell type and decreased in the diabetic heart, while the CMs were increased (Fig. 1D).

### Reclustering of CMs

**Fig. 2:**
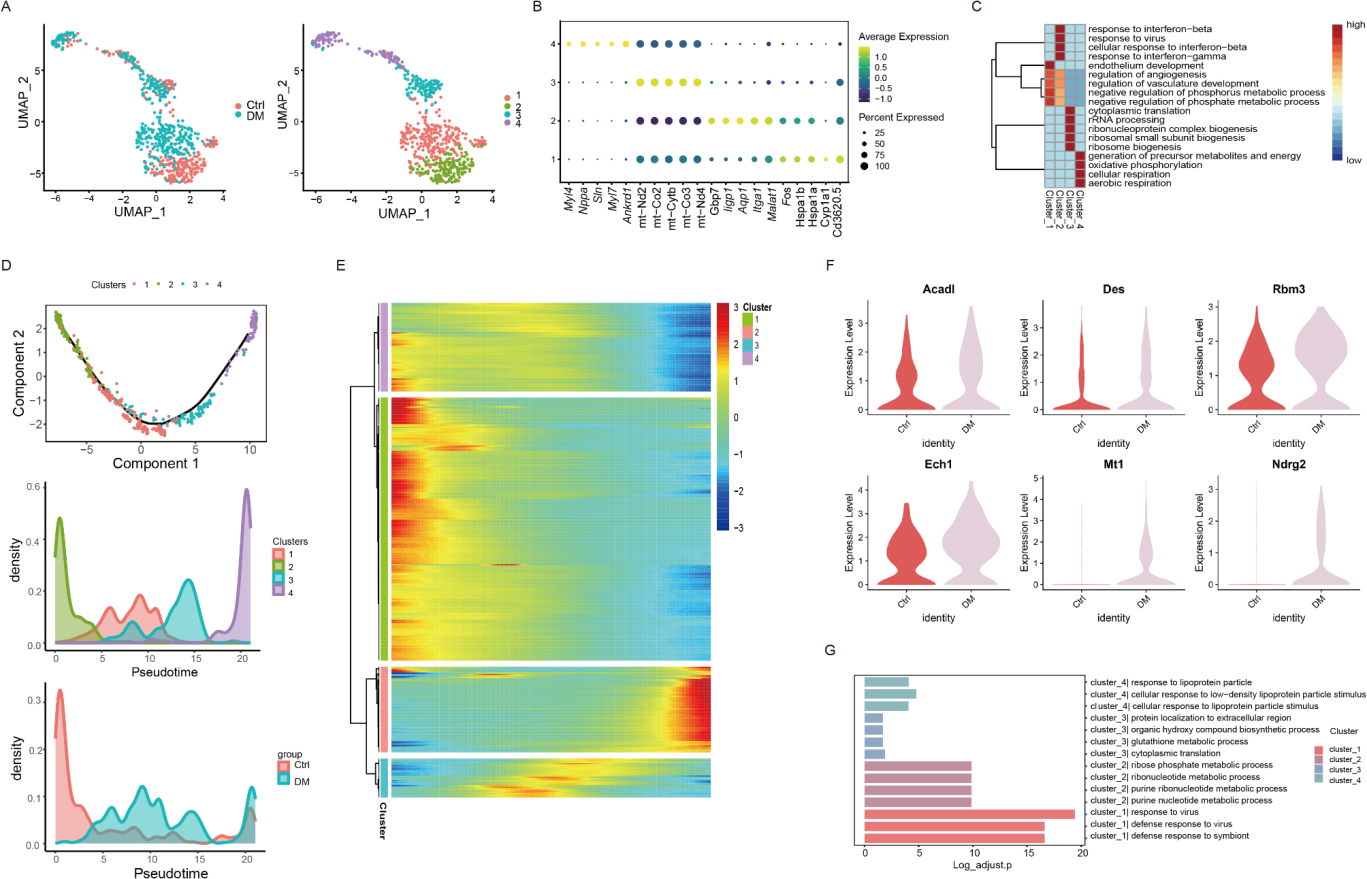
Reclustering of the CMs. (**A**) The CMs were reclustered into four subclusters. (**B**) Highly expressed genes in each cell subpopulation. (**C**) Gene ontology enrichment analysis of the four subclusters of CMs. (**D**) Trajectory analysis showing the differentiation trajectory of the CMs. (**E**) Differentially expressed genes along the pseudotime were grouped into four gene clusters. (**F**) The genes in Cluster 2 were markedly upregulated in the diabetic group. (**G**) Gene ontology enrichment analysis of the four gene clusters

The CMs were reclustered into four subclusters (Fig. 2A) according to the highly expressed gene markers (Fig. 2B). Functional enrichment analysis showed, Cluster 1 was associated with endothelium development, Cluster 2 was relevant to the response to interferon and virus. Cluster 3 was associated with ribosome synthesis and cytoplasmic translation. Cluster 4 was related to cell respiration and energy metabolism(Fig. 2C). The differentiation trajectory depicted that the CMs were differentiated from Cluster 2 to Cluster 1, then to Cluster 3, and finally to Cluster 4 along the pseudotime, accompanied by the transition from the control condition to the diabetic condition as well (Fig. 2D). The differentially expressed genes along the pseudotime were grouped into four gene clusters according to their similar expression dynamics (Fig. 2E) and Acadl, Des, Rbm3, Ech1, Mt1, and Ndrg2 in Cluster 2 were markedly upregulated along the pseudotime (i.e., from the control condition to the diabetic condition) (Fig. 2F).GO enrichment analysis was performed (Fig. 2G) that differential genes were mainly enriched in virus response, ribonucleotide metabolism, cytoplasmic translation and lipoprotein granule response.

### Reclustering of ECs

**Fig. 3:**
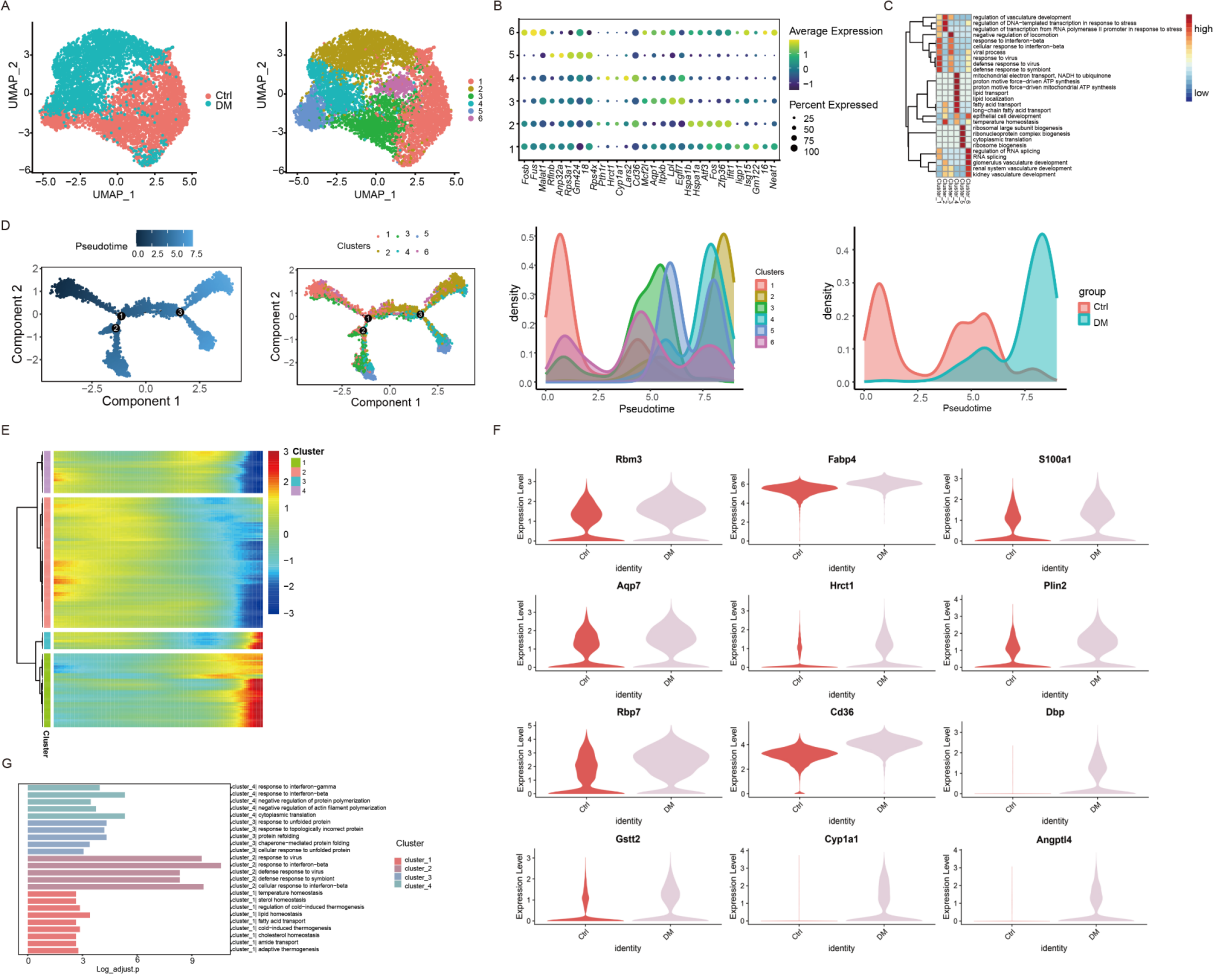
Reclustering of the ECs. (**A**) The ECs were reclustered into six subclusters. (**B**) Highly expressed genes in each cell subpopulation. (**C**) Gene ontology enrichment analysis of the six subclusters of the ECs. (**D**) Trajectory analysis showing the differentiation trajectory of the ECs. (**E**) The differentially expressed genes along the pseudotime were grouped into four gene clusters. (**F**) The genes in Cluster 1 were markedly upregulated in the diabetic group. (**G**) Gene ontology enrichment analysis of the four gene clusters

The ECs were reclustered into six subclusters (Fig. 3A) according to the highly expressed gene markers (Fig. 3B). The gene ontology enrichment analysis revealed that the subclusters of the ECs were enriched in response to the virus and response to interferon (Clusters 1 and 3), vasculature development (Clusters 2 and 6), ATP synthesis and lipid localization (Cluster 4), and the ribosome biogenesis and cytoplasmic translation (Cluster 5) (Fig. 3C). The complex differentiation trajectory of the ECs along the pseudotime (control to diabetic) was principally started with Cluster 1 and divided into four branches, which ended with Clusters 5, 2, and 4, respectively (Fig. 3D). The differentially expressed genes along the pseudotime were grouped into four gene clusters(Fig. 3E), and GO analysis (Fig. 3G) showed that the genes in Cluster 1 were primarily linked to lipid homeostasis and Rbm3, Fabp4, S100a1, and Angptl4 are significantly upregulated in the diabetic heart, (Fig. 3F). Besides, Cluster 2 were linked to the response to the virus, Cluster 3 were relevant to protein folding, and Cluster 4 were associated with cytoplasmic translation and the response to the interferon (Fig. 3G).

### The dynamic cell communication patterns

**Fig. 4:**
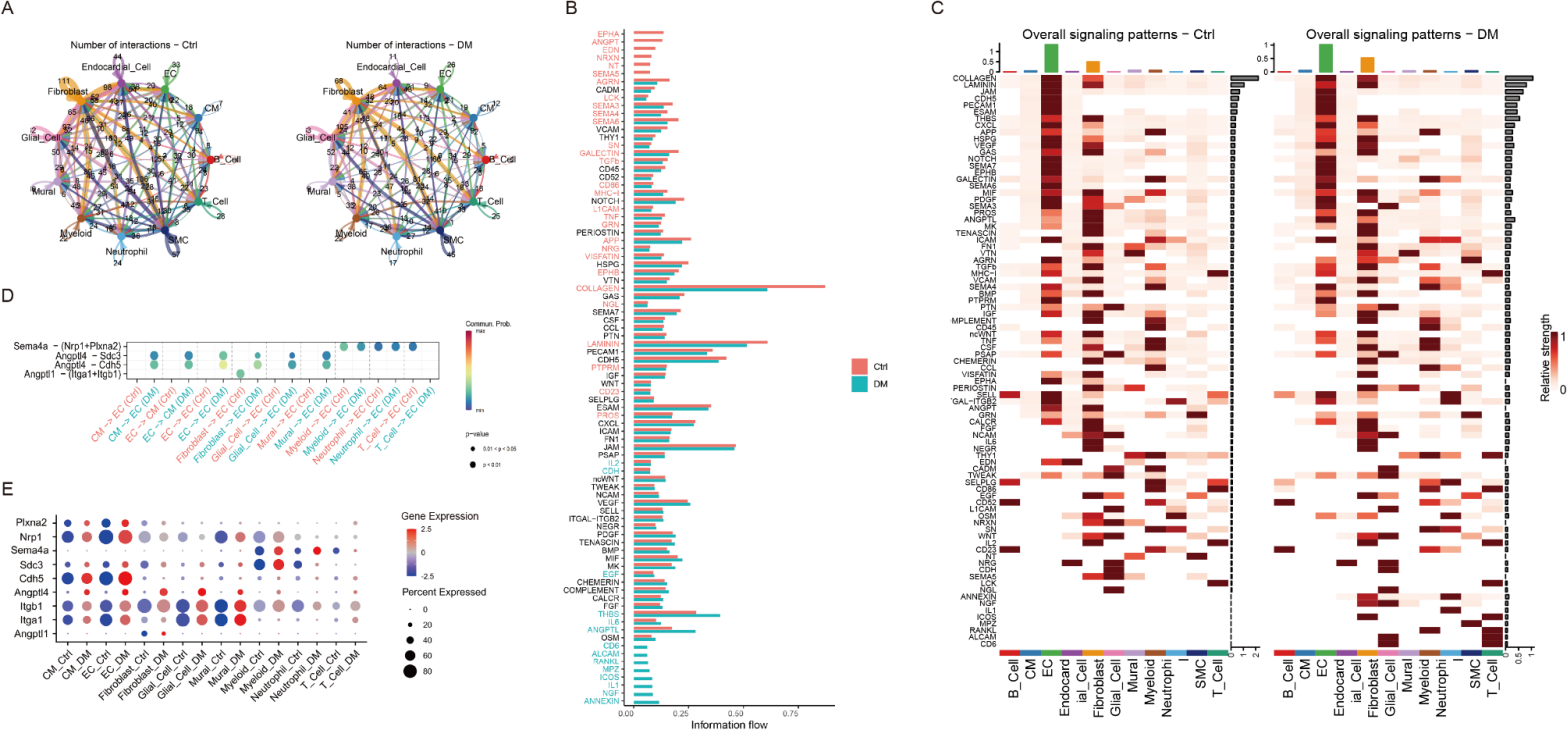
The dynamic cell communication patterns in the control and diabetic hearts. (**A**) The altered cell communication networks in the diabetic heart. (**B**) The information flow for the overall signaling pathways in the control and diabetic hearts. (**C**) The overall signaling patterns of the cells in control and diabetic hearts. (**D**) Significant changes in ANGPTL and SEMA4 signaling in receptor-ligand weight in DM. (**E**) Significantly altered genes in ANGPTL and SEMA4 signaling pathways were expressed in DM

The cellular communication network was altered in the diabetic heart (Fig. 4A). The multiple signaling pathways revealed a similar flow between the control and diabetic hearts, such as the VEGF, PDGF, and WNT signals. In the diabetic heart, the ANNEXIN, NGF, IL1, and ICOS signals were turned on, ANGPTL and THBS signals were increased, NRXN and AGRN signals were decreased, they may attend to the disease progression (Fig. 4B). The detailed changes in the overall signaling patterns of the cells were compared between control and diabetic hearts. The ECs dominated the overall signaling and notably increased the ANGPTL and SEMA4 signals in the diabetic heart compared with the control. (Fig. 4C). Otherwise, four significant ligand-receptor pairs participate in the communication between the ECs and other cell types, such as Angptl1-(Itga1 + Itgb1), Angptl4-Sdc3, Angptl4-Cdh5, Sema4a-(Nrp1 + Plxna2). The ligand Angptl4 and its receptors Cdh5 and Sdc3 are mainly involved in the signaling from the CMs to the ECs (Fig. 4D and E).

### Validation for the novel genes

**Fig. 5:**
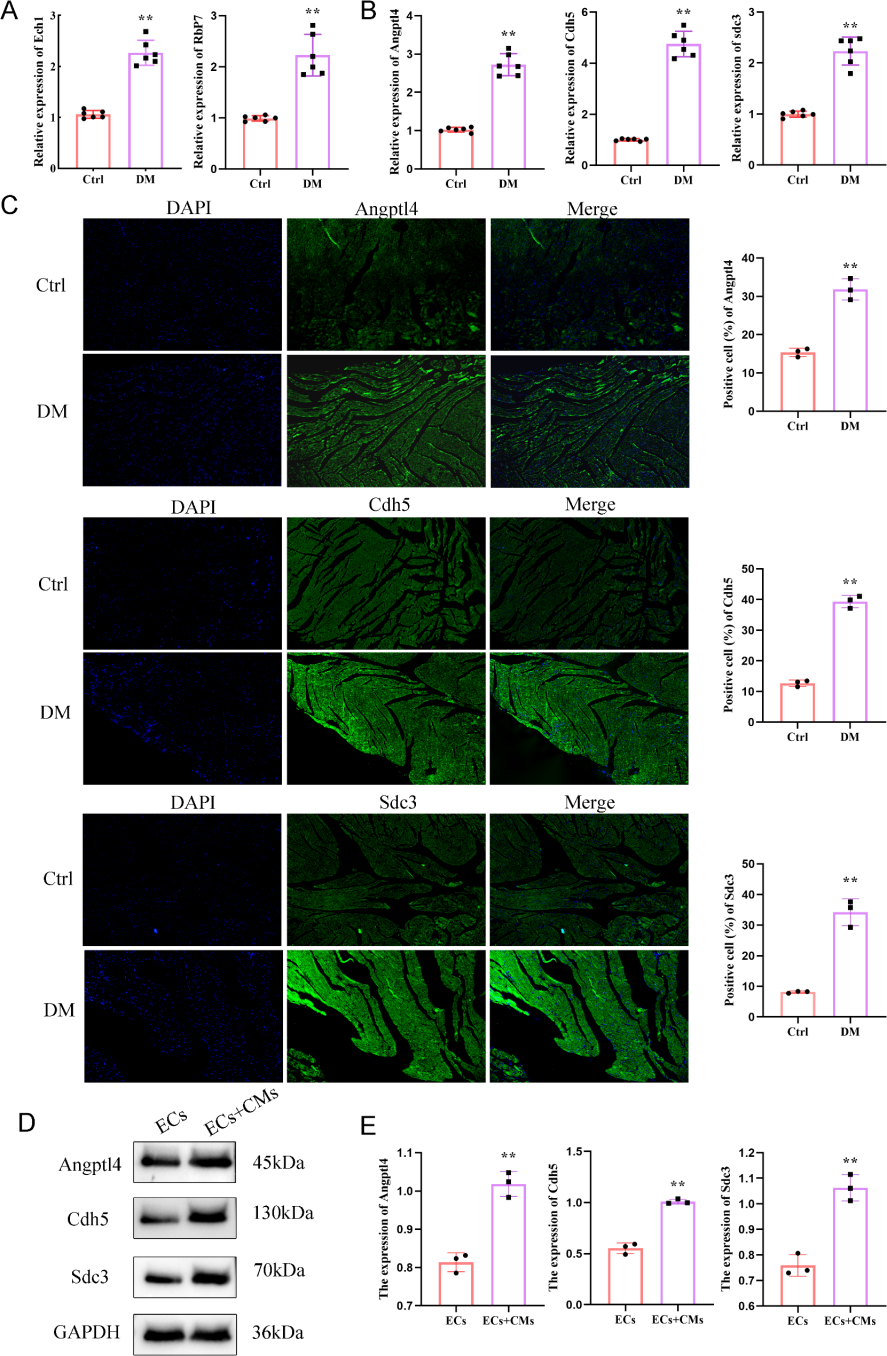
Validation for novel genes. (**A**) The novel genes identified using cell communication by applying both qRT-PCR. *n* = 6, **P < 0.01. (**B**-**C**) PCR (*n* = 6) and immunofluorescence (*n* = 3) were used to detect the expression of Angptl4, Cdh5 and Sdc3 in the myocardium of diabetic mice. **P < 0.01. (**D**-**E**) Western blot was used to detect the protein level of Angptl4, Cdh5 and Sdc3 in endothelial cells under high glucose conditions and endothelial cells co-cultured with cardiomyocytes. *n* = 3 **P < 0.01

It is consistent with the analysis results, the qRT-PCR results revealed the same trend of CMs subpopulation differential genes ECH1 and ECs subpopulation differential genes RBP7 that were up-regulated in the heart that was induced by DM (Fig. 5A ). We performed qRT-PCR and immunofluorescence to detect important ligand-receptors associated with cell communication in the heart as induced by DM. As shown in Fig. 5B and C, we found that Angptl4, Cdh5, and Sdc3 were upregulated in DM, which may act as important regulatory roles in the development of DM. At the same time, we found that the expression of Angptl4, Cdh5 and sdc3 was significantly increased in ECs co-cultured with CMs under high glucose condition compared with ECs cultured alone, indicating that receptor ligands of ANGPTL4-CDH5 and ANGPTl4-SDC3 were involved in the interaction between ECs and CMs under high glucose (Fig. 5D-E).

## Discussion

In the study, we comprehensively analyzed the heterogeneity of cardiomyocytes and endothelial cells in the hearts of diabetic mice, as well as their different differentiation tracks and cell communication characteristics.

We found that ECs and CMs were significantly altered in diabetic. ECs and CMs play an important role in cardiac homeostasis and cardiomyocyte function. ECs and CMs are not only involved in the pathophysiological process of drug-induced cardiotoxicity [13]; abnormal autophagy of ECs and CMs triggered by high glucose can also lead to oxidative stress, endothelial dysfunction and aggravation of inflammatory environment [14]. Chen et al. studies have shown that inhibiting iron death of ECs can improve ECs permeability and protect against cardiovascular injury caused by diabetes [15]. At the same time, signaling pathways in cardiomyocytes and endothelial cells can also be targeted by dapagliflozin (DAPA) in the treatment of type 2 diabetes to reduce cellular stress and inflammation [16].

Single-cell cluster analysis showed that diabetic cardiac ECs were significantly enriched in lipid metabolism and ATP synthesis and regulation of angiogenesis, it is suggested that metabolic reprogramming and compensatory angiogenesis exist in endothelial cells in diabetic hearts. Our analysis and experimental results also show that the expression of Fatty acid binding protein 4 (FABP4) and Retinol binding protein 7 (RBP7) is upregulated in ECs. FABP4 is up-regulated in diabetic hearts and can increase intracellular lipid accumulation, leading to reduced glucose uptake and impaired insulin signaling pathways [17]. Rbp7 is also a member of the fatty acid binding protein family, which can promote fat production and participate in the expression of genes related to retinol metabolism [18], and the disorder of retinol metabolism also promotes the development of diabetic cardiomyopathy [19]. The mechanism of its direct and specific role in lipid metabolism and diabetes needs further research. Notably, the viral defense response enriched in Cluster 1/3 May be due to the innate immune response triggered by endogenous retroviruses in the diabetic heart [20].

The CMs were enriched in the cellular energy metabolism processes, implying the changes in the CMs metabolism in diabetes. CMs accompanied by sustained upregulation of FA oxidation genes such as Acadl and Ech1 with the development of diabeteic. FA is the top choice of energy substrates for CMs and depends on the exogenous supply due to the limited FA synthetization in CMs. Following diabetes, the ECs decreased impairs the uptake of cardiac glucose, resulting in the transformation of energetic metabolism in the heart by shifting toward the exclusive use of FA to satisfy the demand for energy. However, in the long-term, the excessive FA delivery to the CMs impacts unfavorably on cardiac function, such as lip toxicity [21]. In endothelial cells with high glucose exposure, glucose uptake decreased, and high glucose concentrations induced ROS mass production and inhibited glycolysis [9]. As a result, excess glucose is diverted to the auxiliary metabolic pathway of glycolysis, overproducing advanced glycosylation end products, leading to ECs and CMs dysfunction, and promoting the formation and development of diabetic cardiomyopathy [22]. 

The communication between cardiomyocytes enables them to modulate each other’s activity and function. Xujun Wang et al. found that micoRNA secreted from cardiomyocytes can promote the transformation of cardiac fibroblasts into myofibroblasts [23]; we found that the ANGPTL and SEMA4 signals were increased in cardiac ECs under the diabetic condition, wherein the ligand Angptl4 was involved in the EC-CM communication in a paracrine manner. Meanwhile, the expression level of Angptl4 related ligands in ECs co-cultured with CMs was higher than that in ECs under high glucose conditions. Angptl4 is a member of the angiopoietin-like protein family. Angptl4 plays a role in the regulation of TG homeostasis and lipid metabolism by suppressing lipoprotein lipase (LPL) activity [24], and common non-coding variants in LPL gene loci and TGs levels are associated with cardiovascular diseases [25]. LPL catalyzes the hydrolysis of triglyceride (TAG) in glycerol and fatty acid (FA). Knockdown of ANGPTL4 accelerates the catabolism of TRL and increases the oxidation and uptake of FA in brown adipocytes (BAT), At the same time, it can regulate diabetes by regulating glucose metabolism [26, 27]. Therefore, it can be speculated that ANGPTL4 may regulate the cell communication (exp: increased of FA) of ECs-CMs caused by hyperglycemia and become a potential therapeutic target for diabetic cardiomyopathy by regulating lipid metabolism and glucose metabolism. The specific mechanism needs to be further studied.

There were inevitably several limitations in our work. Single cell sequencing analysis has been used as an effective tool to define disease conditions, but this study only stays in the theoretical analysis stage, and cell experiments and animal tissue samples are used for simple verification. The experimental methods and the number of experimental animals we used in our validation work were relatively limited, which might lead to the limited clinical significance of our research results, and the specific deep mechanism needs to be further studied. At the same time, it is necessary to note that the database we analyzed and the experimental validation we conducted in our study all utilized samples from mouse models rather than humans. Studies utilizing the STZ-induced mouse model clearly cannot be completely equated with studies on human diabetes. Therefore, it will still be warranted to conduct single cell sequencing and analysis on samples collected from diabetic patients in clinical settings in future research. To further understands the mechanism of diabetic cardiovascular complications and implement precise treatment; deeper explorations are still requisite.

## Conclusion

In conclusion, CMs and ECs exhibit significant heterogeneities and their metabolism processes are affected in diabetes, and Angptl4 may participate in the cell communication between ECs and CMs by regulating lipid metabolism.

## Data Availability

The datasets used and/or analyzed during the current study are available from the corresponding author upon reasonable request.
